# Effects of disturbed blood flow during exercise on endothelial function: a time course analysis

**DOI:** 10.1590/1414-431X20155100

**Published:** 2016-02-23

**Authors:** F.M. Paiva, L.C. Vianna, I.A. Fernandes, A.C. Nóbrega, R.M. Lima

**Affiliations:** 1 Faculdade de Educação Física, Universidade de Brasília, Brasília, DF, Brasil; 2 Laboratório de Ciências do Exercício, Universidade Federal Fluminense, Niterói, RJ, Brasil

**Keywords:** Blood flow restriction, Vascular function, Flow-mediated dilation, Shear stress

## Abstract

This study aimed to examine the time course of endothelial function after a single handgrip exercise session combined with blood flow restriction in healthy young men. Nine participants (28±5.8 years) completed a single session of bilateral dynamic handgrip exercise (20 min with 60% of the maximum voluntary contraction). To induce blood flow restriction, a cuff was placed 2 cm below the antecubital fossa in the experimental arm. This cuff was inflated to 80 mmHg before initiation of exercise and maintained through the duration of the protocol. The experimental arm and control arm were randomly selected for all subjects. Brachial artery flow-mediated dilation (FMD) and blood flow velocity profiles were assessed using Doppler ultrasonography before initiation of the exercise, and at 15 and 60 min after its cessation. Blood flow velocity profiles were also assessed during exercise. There was a significant increase in FMD 15 min after exercise in the control arm compared with before exercise (64.09%±16.59%, P=0.001), but there was no change in the experimental arm (-12.48%±12.64%, P=0.252). FMD values at 15 min post-exercise were significantly higher for the control arm in comparison to the experimental arm (P=0.004). FMD returned to near baseline values at 60 min after exercise, with no significant difference between arms (P=0.424). A single handgrip exercise bout provoked an acute increase in FMD 15 min after exercise, returning to near baseline values at 60 min. This response was blunted by the addition of an inflated pneumatic cuff to the exercising arm.

## Introduction

Exercise training reduces cardiovascular risk, preventing primary (1) and secondary (2 3 24) cardiovascular events. Modulation of cardiovascular risk factors is considered to be the main mechanism explaining the beneficial effect of exercise. However, recent studies have demonstrated that established risk factors do not account for the total reduction in cardiovascular risk associated with exercise training (3,5). Therefore, improvement in vasculature structure and function resulting from training may play an important role in a reduction of cardiovascular risk (6). Conversely, impaired endothelial function is associated with development of atherosclerosis and increased risk of future cardiovascular events (7 8 9-10). An increase in flow-mediated dilation (FMD), a measure of endothelial function, has been reported after exercise training with varied exercise modalities and in different populations ([Bibr B11]11^12^-^13^). Some studies have reported a significant improvement in FMD after only 2 weeks of exercising (14,15).

However, an increasingly popular training method known as Kaatsu training or vascular occlusion training has been shown to hinder chronic adaptations in endothelial function associated with exercise (15,16). A recent study reported a 30% decrease in FMD after 4 weeks of handgrip training combined with this training method (12). Placement of an inflated cuff on the exercising limb causes blood flow restriction (BFR) to the exercising skeletal muscle and decreases venous return. This leads to increases in muscle mass and strength similar or greater than traditional training (12,17). However, BFR significantly impairs the increase in blood flow velocity and shear rate observed during exercise (18). An increase in blood flow velocity and shear rate are the main physiological stimuli for the adaptations in endothelial function induced by exercise training (15,18,19). An increase in mean and antegrade shear rates is associated with beneficial adaptations in vascular endothelial structure and function (20,21), and it provides an important stimulus for vasodilation of conduit arteries during exercise (22). In addition, inflation of a pneumatic cuff promotes an increase in retrograde shear rate in a dose-dependent manner. This component of blood flow is considered to be detrimental to the endothelium and may hinder the beneficial adaptations in response to exercise (12,23,24).

Acute increases in endothelial function after a single exercise bout are also affected by BFR. Studies that have reported a significant increase in FMD immediately after cessation of exercise have demonstrated that addition of BFR inhibits the acute response in the cuffed arm only (15,18). Higher FMD values have also been observed 1 h after a single exercise bout in different modalities (25,26). Although these above-mentioned studies showed the FMD response to a single exercise bout, the time course of endothelial function after exercise combined with BFR is still unclear. Considering that adaptations may result from temporal summation of acute responses (27), understanding the effects of exercise on FMD in a time-dependent manner is important.

Therefore, this study aimed to examine the time course of endothelial function after a single handgrip exercise bout combined with BFR in healthy young men. We hypothesized that modulation of the blood flow shear rate profiles caused by BFR during exercise is detrimental to the acute effects of exercise on endothelial function.

## Material and Methods

### Subjects

Nine healthy young men (means±SD: age=28±5.8 years) from the student body of Universidade Federal Fluminense (Brazil) were selected to participate in the study. All of the subjects were recreationally active. Exclusion criteria were as follows: diagnosis or evidence of any cardiovascular, metabolic, orthopedic, neurological or endocrine disease that knowingly affects endothelial function; use of any medication that can interfere with cardiovascular function; and a risk of adverse response to exercise. The study procedures were approved by the Ethics Committee of the Universidade Federal Fluminense and adhered to the Declaration of Helsinki. Written informed consent was obtained from all of the individual participants included in the study.

### Experimental design

Subjects underwent a single testing session in which all of the experimental procedures were conducted. Before reporting to the laboratory, subjects were asked to fast and refrain from caffeine, tobacco, alcohol, and strenuous physical activity for at least 12 h before the experiment.

Initially, FMD and blood flow shear rate profiles were assessed bilaterally after resting for 20 min in the supine position in a quiet, temperature-controlled room. Subsequently, a 20-min handgrip exercise bout was performed and FMD was reassessed 15 and 60 min after cessation of the exercise. In addition, a cuff was inflated to 80 mmHg throughout the duration of the exercise in one of the arms to induce changes in the blood flow shear rate patterns. Blood flow shear rate profiles were also assessed before and during the handgrip exercise.

### Handgrip strength

Two digital hand dynamometers (MLT004/ST Grip Force Transducer, ADInstruments, New Zealand) that were connected to a data acquisition system (PowerLab, ADInstruments) were used for the assessment of handgrip strength. For all strength measurements, the subjects were in the supine position with arms by their side and elbows fully extended. To determine handgrip strength, the subjects performed three maximal voluntary contractions (MVCs) with each arm. They were asked to grip the dynamometer with maximal effort for 3 s, repeating this procedure three times for each arm with 1-min interval between trials. The left arm was tested first in all subjects. LabChart (ADInstruments) was used to analyze the obtained data, and the median of all trials for each arm was considered the MVC.

### Endothelial function and blood flow shear rate profiles

Bilateral imaging of the brachial artery was conducted by two experienced ultrasonographers in accordance with the International Brachial Artery Reactivity Task Force Guidelines, also taking in consideration recent updates to the methodology (28,29). All of the images were obtained using the LOGIC P5 (GE Healthcare, United Kingdom) and the VIVID 7 (GE Healthcare) Doppler ultrasound with multi-frequency linear array transducers set to 10 and 5 MHz was used for the analysis of diameters of vessels and blood flow velocity, respectively. The images were obtained in the longitudinal view approximately 2 cm proximal to the cubital fossa. Gain settings were adjusted to allow for an optimal view of the anterior and posterior intimal interfaces of the artery. Doppler velocity profiles were collected simultaneously using a pulsed signal at a corrected insonation angle of 60° to the vessel with the velocity cursor positioned mid artery to sample the volume.

FMD consisted of an initial 1-min recording of the vessel diameter and velocity profiles at rest. The resting period was followed by a vascular occlusion, involving inflation of two pneumatic cuffs that were positioned in both arms approximately 2 cm distal from the cubital fossa to 250 mmHg, for 5 min via a rapid cuff inflator (E20 Rapid Cuff Inflator; D.E. Hokanson, USA). Images for the vessel diameter and velocity profiles were then continuously obtained from the final 30 s of occlusion until 2 min after the release of the cuff. All images were captured by an USB video board (Easy Cap, Leadership, Brazil) at a frequency of 30 Hz and then saved on an external hard drive for posterior offline analysis.

Examination of the blood shear rate profiles was performed in a subset of the sample (n=7) before and during the handgrip exercise. At both time points, the cuff that was previously positioned in the experimental (EXP) arm was insufflated at 80 mmHg. The purpose of this experiment was to assess the effect of restriction of blood flow and exercise on blood shear rate patterns. Images were recorded for 1 min in both arms and saved on an external hard drive for posterior analysis.

### Data analysis

The brachial artery diameters and blood flow velocity profiles were analyzed using a semi-automated edge-detecting software (FMD Studio; Institute of Clinical Physiology, Italy). The reproducibility of this software was previously demonstrated elsewhere (30). Arterial diameters were calculated as the distance between the anterior and the posterior walls at the blood vessel interface. The resting diameter was defined by the average of 1 min of data that were obtained after a minimum of 20 min in the supine position at rest in a quiet, temperature-controlled room. The peak diameter was defined as the largest diameter that was achieved after release of the occluding cuff. Brachial artery FMD was calculated as the percent change in vessel diameter from rest to peak diameter.

The antegrade and retrograde components of blood flow were defined as the area of tracing above and below 0 cm/s from the Doppler ultrasound scale, respectively. The mean shear rate was calculated as the difference between the antegrade and the retrograde shear rate components. The shear rate area under the curve (AUC_SR_) was calculated from release of the occluding cuff until the peak diameter was achieved; this variable was later used for normalization of FMD values (31). The oscillatory shear index was defined as follows: |retrograde shear|/(antegrade shear + |retrograde shear|) (22). Values for oscillatory shear ranged from 0 to 0.5, where 0 corresponds to the unidirectional shear rate and a value of 0.5 represents pure oscillation. Blood flow in the brachial artery was obtained using the formula proposed by Pennati et al. (32).

### Exercise bout

The exercise bout consisted of 20 min of bilateral handgrip with a resistance of 60% MVC that was performed at a rate of 15 contractions per min (one contraction every 4 s) alternating between both arms. An electronic metronome was used to determine the pace. The subjects were in the supine position with arms by their side and elbows fully extended. Visual feedback was projected at the ceiling to ensure that the work intensity was maintained throughout the duration of the exercise. An optional 1-min rest was allowed after 10 min of exercising. The resting time did not count towards the 20-min total exercise duration. Rates of perceived exertion were assessed after 10 min and immediately after cessation of the exercise using a 0-10 modified Borg scale. For one of the arms, which was randomly selected for all subjects, the pneumatic cuff that was previously placed around the forearm was insufflated to 80 mmHg before initiation of the exercise, and maintained for the whole duration (EXP). The contralateral arm cuff remained at 0 mmHg (CON).

### Statistical analysis

Statistical analyses were performed using the Statistical Package for Social Sciences version 22.0 for Mac (SPSS Inc., USA). The Shapiro-Wilk test was used to verify normality of the data. To determine the acute effects of the exercise bout on endothelial function, a two (EXP and CON arms) by three (baseline, 15, and 60 min) repeated-measures analysis of variance (ANOVA) was performed. Subsequent ANOVAs were performed to analyze the acute effects on brachial artery diameters, time to peak dilation, AUC_SR_, and blood flow. Differences between means were evaluated using the *post hoc* LSD test. The Friedman test with the Wilcoxon signed-rank post hoc test was used to analyze the acute effects of the exercise bout for non-parametric data. Paired *t*-tests were performed to determine differences in shear rate between the CON arm and EXP arm at rest and during handgrip exercise. Pearson correlations were used to examine the relation between shear rates during exercise and the change in FMD, as well as that between oscillatory shear index during exercise and changes in FMD. Adequate sample size was calculated by computing the achieved power for a within-factor 2×3 ANOVA. The input parameters were alpha (0.05), sample size (n=9), and effect size (partial eta-squared: 0.476). Therefore, the observed power was 0.882, indicating an adequate sample size. Data are reported as means±SE or means±SD. Statistical significance was set at P≤0.05.

## Results

Baseline characteristics of the subjects are shown in Table 1. All of the subjects successfully completed the exercise bout. Rates of perceived exertion were 4.56±0.65 and 5.38±0.53 at 10 and 20 min, respectively.

**Table:**
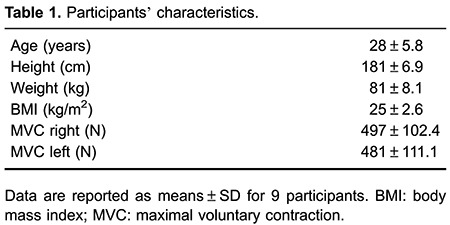

### Endothelial function and blood flow shear rate profile

Values for vascular diameters, time to peak dilation, AUC_SR_, and blood flow are shown in Table 2. Results from the 2×3 ANOVA showed no significant interaction for AUC_SR_ (P=0.930), but there was a significant main effect for time (P=0.005). Post hoc analysis showed an increased AUC_SR_ 15 min after cessation of exercise compared with baseline in the CON (P=0.007) and EXP (P=0.027) arms. Similarly, there was no significant interaction for peak diameter (P=0.597), but there was a significant main effect for time (P=0.050). Post hoc analysis showed a significant increase in peak diameter compared with baseline values only in the CON arm at 15 and 60 min after the exercise bout (P=0.007 and P=0.014, respectively). There were no significant interactions for resting diameter and time to peak dilation (P=0.113 and P=0.511, respectively), neither was there a significant main effect for condition (P=0.061 and P=0.967) or time (P=0.156 and P=0.282) for both variables. There was no significant interaction for blood flow (P=0.191), but there was a significant main effect for condition (P=0.036). *Post hoc* analysis showed a higher blood flow in the EXP arm 15 min after exercise compared with the CON arm (P=0.018).

**Table:**
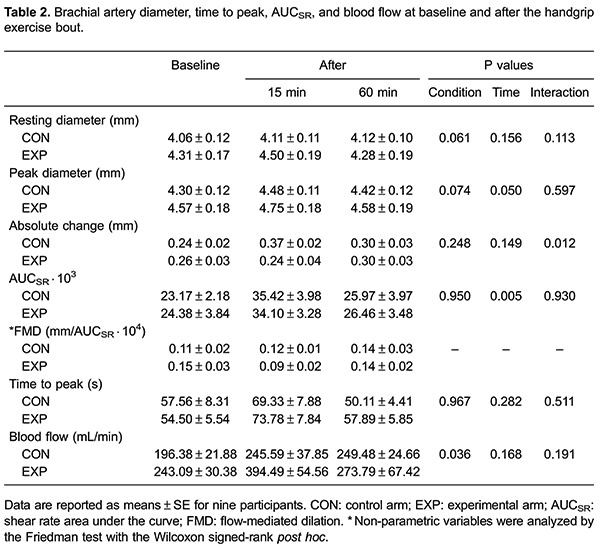

**Figure 1:**
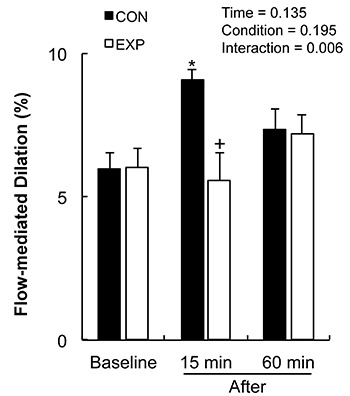
Flow-mediated dilation (FMD) at baseline, 15 and 60 min after a handgrip exercise bout for both arms. Data are reported as mean±SE. EXP: Experimental arm. *P≤0.01 compared to the same arm at rest; ^+^P≤0.01 compared to the control arm (CON) at the same time point (2×3 repeated-measures ANOVA test).

**Figure 2:**
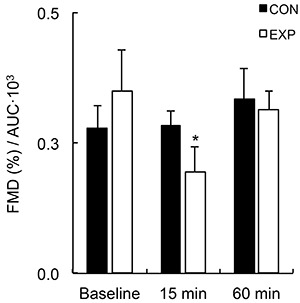
Flow-mediated dilation (FMD)/shear rate area under the curve (AUC_SR_) at baseline and after (15 and 60 min) a handgrip exercise bout for both arms. Data are reported as mean±SE. EXP: experimental arm. *P≤0.05 compared to the control arm (CON) at the same time point (2×3 repeated-measures ANOVA test).

The results from the 2×3 ANOVA for FMD are shown in Figure 1. There was no difference in baseline FMD between both arms (CON arm=5.98±0.56%; EXP arm=6.04±0.63%, P=0.478). There was a significant time × condition interaction for FMD (P=0.006). Post hoc analysis showed that FMD was increased 15 min after the exercise bout in the CON arm compared with baseline (baseline=0.24±0.02 mm to 15-min post-exercise=0.37±0.02 mm; percent change=64.09%±16.59%, P=0.001), while there was no change in the EXP arm (baseline=0.26±0.03 mm to 15-min post-exercise=0.24±0.04 mm; percent change=-12.48±12.64, P=0.252). FMD values at 15 min post-exercise were significantly higher for the control arm in comparison to the experimental arm (P=0.004). FMD values at 60 min after exercise were similar to those at baseline, with no significant difference between both arms (P=0.424). After FMD (%) was normalized by AUC_SR_ (Figure 2), FMD values on the CON arm remained similar throughout 60 min (P=1.000), while there was a significant decrease in the EXP arm (P=0.050). *Post hoc* analysis showed a significantly lower FMD/AUC_SR_ in the EXP arm 15 min after the exercise bout compared with rest (P=0.011).

**Figure 3:**
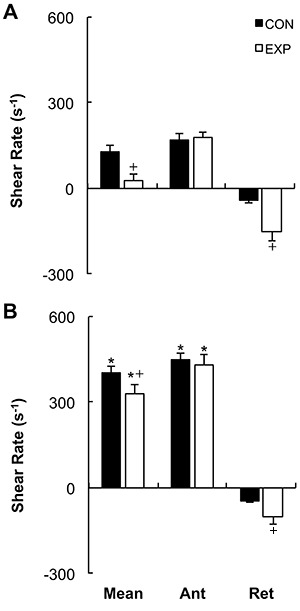
Shear rate profiles at rest and during handgrip exercise for control (CON) and experimental (EXP) arms. *A*, Rest, with the cuff inflated to 80 mmHg in the EXP arm. *B*, During exercise, with the cuff inflated to 80 mmHg in the EXP arm. Data are reported as mean±SE. Ant: antegrade, Ret: retrograde. *P≤0.01 compared to the same arm at rest; ^+^P≤0.01 compared to the CON arm at the same time point (2×3 repeated-measures ANOVA test).

**Figure 4:**
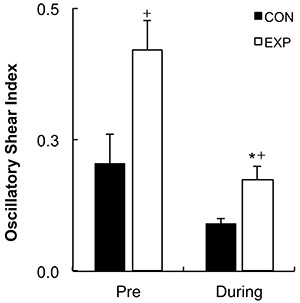
Oscillatory shear index at rest (Pre) and during handgrip exercise for control (CON) and experimental (EXP) arms. Data are reported as mean±SE. *P≤0.01 compared to the same arm at rest; ^+^P≤0.01 compared to the CON arm at the same time point (2×3 repeated-measures ANOVA test).

**Figure 5:**
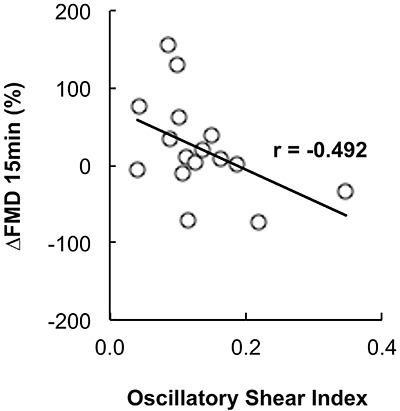
Pearson’s correlation between change in flow-mediated dilation (ΔFMD) at 15 min and the oscillatory shear index.

Figure 3 shows the results of the blood flow shear rate profile immediately before and during the exercise bout. There was a significant increase in the retrograde shear rate component upon inflation of the cuff in the EXP arm (pre-inflation=-28.99±11.69/s to post-inflation=-153.18±30.82/s, P=0.001). Consequently, the retrograde component was significantly higher in the EXP arm than in the CON arm before initiation of exercise (CON arm=-42.05±-9.61/s and EXP arm=-153.18±-30.82/s; P=0.007). Mean shear rate at rest was significantly higher in the CON arm than in the EXP arm (CON arm=126.82±26.27/s and EXP arm=26.80±23.81/s, P=0.023). The increased retrograde flow shear rate and the decreased mean shear rate remained significantly different for the EXP arm during the exercise bout (P=0.044 and P=0.033, respectively). Mean and antegrade shear rates during exercise were significantly increased compared with rest in both the CON (P<0.001) and EXP arms (P<0.001), but there was no significant increase in retrograde shear rate (CON arm P=0.411, EXP arm P=0.098). Oscillatory shear rate was significantly higher in the EXP arm in comparison to the CON arm at rest upon inflation of the cuff (P=0.034) and during handgrip exercise (Figure 4, P=0.006). Shear rate oscillation in the EXP arm was significantly lower during the exercise when compared to rest (P=0.005). There was no significant correlation between shear rates during exercise and the changes in FMD. However, a significant negative correlation was found between the oscillatory shear index during exercise and changes in FMD at 15 min after exercise (r=-0.492; P≤0.05, Figure 5).

## Discussion

The present study examined the acute effects of handgrip exercise combined with BFR on endothelial function of healthy young men. The main findings of the present study were as follows: 1) FMD was increased in the CON arm 15 min after a single handgrip exercise bout, and returned to near baseline values at 60 min; 2) addition of BFR to the EXP arm blunted the increase in FMD after cessation of exercise; 3) BFR in the EXP arm decreased mean shear rate and increased retrograde shear rate before and during the exercise bout; and 4) the oscillatory shear index was significantly higher in the EXP arm at rest and during exercise in comparison to the CON arm; this index was inversely correlated with the percent changes in FMD. Therefore, we established that BFR was detrimental to the acute effects of a single handgrip exercise bout on FMD, which might be explained by the differences in shear rate profiles between arms during exercise.

Chronic increases in endothelial function have been extensively reported in response to exercise training in different populations (11,13,14). However, addition of BFR to the exercising limb has been shown to impair these beneficial adaptations (15). A recent study by Credeur et al. (12) demonstrated a 30% decrease in brachial artery FMD after 4 weeks of dynamic handgrip training combined with BFR, while FMD in the contralateral uncuffed arm increased by 24%. Similar results have been reported in acute studies. A single exercise bout was reported to acutely increase FMD (15,18,26,33), while addition of the BFR training method blunted this response (15,18). Decreased FMD in response to exercise combined with vascular occlusion has also been reported in acute studies; Tinken et al. (18) reported a significant reduction in FMD following a 30-min recumbent bicycle protocol combined with BFR, while the contralateral uncuffed arm showed augmented endothelial function. The present study found a significant increase in FMD after exercise only for the CON arm, while that in the EXP arm did not differ from the baseline measurement at any time point. These findings support those from previous studies that assessed acute FMD responses to exercise. However, in contrast to some studies, there was no reduction in FMD at any time point for the EXP arm. This difference between studies might be explained by differences in the applied exercise protocol or the population of the study (25,33).

A primary objective of the present study was to examine the acute effects of a single exercise bout on endothelial function and blood flow velocity profiles in a time-dependent manner. Johnson et al. (33) reported an increase in brachial artery FMD immediately after a single sub-maximal treadmill exercise bout, with the values returning to near baseline after 1 h. Similar to their study, increased FMD that was observed for the CON arm in the present study was only significant at 15 min, returning to near baseline values after 60 min. However, Atkinson et al. (25) reported no changes in brachial artery FMD immediately after low-intensity (5%, 10%, and 15% MVC) handgrip exercise, while there were significantly higher values at 60 min for the 15% MVC protocol. They concluded that the acute responses to handgrip exercise may be intensity-dependent. Therefore, the difference between studies can be explained by the much higher intensity used in the present study (60% MVC).

With regard to acute changes in blood flow, Bousquet-Santos et al. (34) reported increased forearm blood flow during reactive hyperemia and vascular reactivity for 60 min after a single maximal exercise bout, and flow returned to near baseline values after 120 min. Similarly, Baynard et al. (35) reported an increase in forearm blood flow and vasodilatory capacity after maximal treadmill exercise. In contrast to these previous studies, in our study, there was no significant increase in brachial artery blood flow after the exercise bout in the CON and EXP arms at any time point. AUC_SR_ was increased in the CON and EXP arms 15 min after exercise and returned to near baseline values after 60 min. This finding is in accordance with a recent study, which used a similar exercise protocol with lower intensity (25). However, there are conflicting previous studies that did not report increased AUC_SR_ after a single exercise bout (18,26). In our study, although AUC_SR_, which is a measure of the stimulus to vasodilation, was increased for both arms after the exercise bout, only the CON arm showed a significant increase in FMD. Therefore, the increased shear stimulus observed during reactive hyperemia does not fully explain the changes in endothelial function that were observed in our study. After FMD was normalized by AUC_SR_, a significant reduction in FMD was found 15 min after exercise for the EXP arm only. Normalization of FMD by the total shear stimulus is not yet widely used. However, a recent study described that this index is a better representation of endothelial function than only using conventional FMD values (36). Normalized FMD is also better for discriminating subjects with increased cardiovascular risk (37). A reduction in FMD/AUC_SR_ for the EXP arm indicates a detrimental effect of exercise combined with BFR on endothelial function.

Recent studies have suggested that modulations in the blood flow shear rate profile may be responsible for the acute and chronic changes in endothelial function associated with exercise (12,15,18,21,22). Inflation of a pneumatic cuff during exercise significantly alters the blood flow shear rate profile. This hinders or abolishes the increase in mean and antegrade shear rates observed at the onset of exercise, consequently affecting acute and chronic responses (15,18). These two components of blood flow are considered to provide an important physiological stimulus to the vascular endothelium, and are associated with beneficial adaptations in endothelial function, even in the absence of an exercise stimulus (20,21). Chronic adaptations in endothelial function have been proposed to occur in response to the accumulated effect of episodic increases in antegrade shear rate (38). In contrast to previous reports, in the present study, BFR did not elicit a significant reduction in antegrade shear rate compared with the uncuffed arm, although the pressure to which the cuff was inflated is in accordance with the literature. Despite antegrade shear rate not being affected by BFR, mean shear rate was significantly lower in the EXP arm compared with the CON arm because of the increased retrograde shear rate in the cuffed arm at rest and during exercise. Increased retrograde shear rate in response to a pneumatic cuff has been previously reported (12). Recent studies have shown a significant dose-response relationship between cuff pressure and the magnitude of increase in retrograde shear rate (23,24,39), which were also associated with a significant reduction in FMD values.

A few limitations of our study warrant discussion. First, the number of time points in which post-exercise FMD was assessed was limited to two times. Incorporating more post-exercise FMD measures might have been advantageous, although the main findings would probably be similar to our results. Our study was exclusively restricted to men and thus our findings cannot be extrapolated to women. Additionally, whether post-exercise endothelial function is similarly affected by BFR in women is unknown. Future studies on these issues are warranted.

### Perspectives

A growing body of evidence supports the use of exercise (low-intensity resistance training, walking, and cycling) combined with BFR to enhance hypertrophic and strength responses in skeletal muscle (12,17). However, our data indicate that exercise with BFR abolishes the post-exercise increase in endothelial function. This might be a disadvantage of this method, especially for those with known risk factors or cardiovascular diseases. However, for those with orthopedic injuries and low cardiovascular risk, the use of moderate BFR combined with low-intensity exercise may still be useful.

In conclusion, a single handgrip exercise bout provoked an acute increase in FMD 15 min after exercise, returning to near baseline values at 60 min. This effect was blunted by the addition of an inflated pneumatic cuff to the exercising arm. These results are possibly explained by changes in blood flow profiles observed during exercise with BFR, especially an increased retrograde shear rate and reduced mean shear rate. The number of time points in which FMD was assessed is a possible limitation to the study design. Therefore, future studies using a higher number of time points should be conducted to further understand the time course effects of a single exercise bout with BFR.
